# Association between person-centered care during pregnancy and perinatal depression in Ghana

**DOI:** 10.1186/s12884-025-07966-6

**Published:** 2025-10-06

**Authors:** Lakshmi Gopalakrishnan, Raymond A. Aborigo, Christopher A. Ayuure, Patience A. Afulani

**Affiliations:** 1 Institute for Global Health Sciences, University of California, San Francisco, CA USA; 2 Navrongo Health Research Centre, Post Office Box 114, Navrongo, Ghana; 3 Department of Obstetrics, Gynecology, and Reproductive Sciences, University of California San Francisco, San Francisco, CA USA; 4 Department of Epidemiology & Biostatistics, University of California San Francisco, San Francisco, CA USA

**Keywords:** Postpartum depression, Postnatal depression, Sub-Saharan Africa, Maternal health, Respectful maternity care, Quality of care, Maternal mental health

## Abstract

**Background:**

Risk factors for perinatal depression (PND) have been well documented, yet the relationship between person-centered care during antenatal and childbirth care and PND remains understudied.

**Objective:**

To examine the association between person-centered antenatal care (PCANC) and person-centered maternity care (PCMC) and PND.

**Study Design:**

Data are from cross-sectional surveys with 293 postpartum women in Ghana. The 10-item Edinburgh Postnatal Depression Scale (EPDS), together with validated 36-item PCANC and 30-item PCMC scales were administered to participants. The PCANC and PCMC scale both have 3 subscales: dignity and respect, communication and autonomy, and supportive care. Bivariate and multivariable logistic regressions were used to examine the relationship between PCANC and PCMC and PND measured within six months postpartum.

**Results:**

In bivariate analyses, higher PCANC scores were associated with lower odds of perinatal depression (OR: 0.97, 95% CI: 0.95-0.99). After controlling for confounders, this association strengthened (aOR: 0.96, 95% CI: 0.93-0.99). Dignity and respect (aOR: 0.96, 95% CI: 0.94-0.98) and responsive and supportive care subscales (aOR: 0.96, 95% CI: 0.93-0.99) were significantly associated with reduced odds of perinatal depression. While PCMC showed protective trends, only the dignity and respect subscale showed a significant association with lower odds of perinatal depression (aOR: 0.97, 95% CI: 0.95-0.99).

**Conclusion:**

PCANC, particularly its components of dignity and respect and responsive and supportive care, may serve as a protective factor against PND. These results suggest that implementing person-centered approaches could offer a feasible strategy for addressing PND in low-resource settings where routine mental health screening is unavailable.

**Supplementary Information:**

The online version contains supplementary material available at 10.1186/s12884-025-07966-6.

##  Why was this study conducted?

This study was conducted to assess whether person-centered care during antenatal and childbirth care were associated with perinatal depression in Ghana, where routine screening is not available.

### Key findings

Person-centered antenatal care was associated with lower odds of postpartum depression, with each point increase corresponding to a 5% reduction in odds of perinatal depression. The protective effects were specifically linked to dignity and respect and responsive and supportive care components. However, person-centered care during maternity showed no significant association with perinatal depression, except for the dignity and respect subscale.

### What does this add to what is known?

This study provides novel evidence on person-centered care by demonstrating the specific importance of care delivered by providers during antenatal care in protecting against perinatal depression. Specifically, it underscores the importance of dignity, respect, and responsive care during pregnancy for maternal mental health outcomes, offering insights for intervention design.

## Introduction

Maternal mortality and morbidity persist as global health challenges, despite a 38% decline in global maternal mortality during the last decade [1]. An estimated 810 maternal deaths occur daily worldwide, with low and middle-income countries (LMICs) accounting for 94% of these deaths [1]. Sub-Saharan Africa bears the greatest burden, accounting for two-thirds of global maternal deaths [2]. In Ghana, the maternal mortality ratio is 283 per 100,000 live births, still far higher than the global target of 70 per 100,000 live births [3–5]. While substantial efforts have focused on reducing maternal mortality, maternal morbidity, particularly mental health conditions such as perinatal depression, has received much less attention.

For every maternal death, approximately 20 women experience various morbidities, including perinatal depression (PND) [6]. The impact of PND is far-reaching, increasing maternal suicide risk [7–10], impairing mother-infant interactions, and adversely affecting children’s nutritional, psychological, behavioral, and cognitive development [11–21]. The burden of PND is particularly concerning in sub-Saharan Africa, where a recent systematic review reported prevalence rates ranging from 7.6 to 50.6%, with an average of 18.6% [22]. In Ghana specifically, community-based studies have documented postpartum depression rates varying from 3.4 to 33.5% [23–26].

The quality of care women receive during pregnancy and childbirth plays a crucial role in both their physical and mental health outcomes. Poor quality maternal health care contributes to preventable deaths and complications [27, 28], while also impacting women’s mental health during the vulnerable perinatal period. A key dimension of the quality of maternal health care is person-centered maternal health care (PCMHC), which represents a comprehensive approach to maternal healthcare that emphasizes holistic, responsive, and dignified care beyond merely avoiding poor treatment [29]. PCMHC includes person-centered antenatal care (PCANC) and person-centered maternity care (PCMC), emphasizing respectful and compassionate care and recognizing women’s preferences, needs, and values throughout the pregnancy and childbirth experience [30, 31].

PCMHC could influence maternal health outcomes through multiple pathways. The direct pathway operates through improved provider-patient communication, enhanced respect and dignity, and sustained emotional support [29], which is associated with better care delivery, greater patient engagement, and improved psychosocial health [32–35]. Indirectly, these approaches shape women’s healthcare-seeking behaviors - positive experiences with respectful and compassionate care encourages continued engagement with maternal health services post-delivery [28, 36, 37]. Conversely, experiences of poor quality care not only deter individual women from seeking facility-based care but also create broader community-level effects, where negative perceptions discourage other women from accessing facility-based care and delivery services [38, 39].

The perinatal period represents a critical period for maternal mental health interventions. While research has identified risk factors for PND in Ghana [23–26] and globally [40], the relationship between person-centered care during pregnancy and childbirth and PND remains understudied. In Ghana, where routine prenatal mental health screening is unavailable, understanding the relationship between PCMHC and PND is particularly important. Stigma, discomfort in discussing mental health [41] and difficulty distinguishing normal pregnancy-related emotional changes from concerning symptoms create barriers to identifying and addressing perinatal mental health needs. Although prenatal interventions can effectively prevent postpartum depression [42], the perinatal mental health of women in Ghana continues to be undetected and under-treated [43, 44].

The aim of this study is to examine the association between PCANC and PCMC with PND, focusing on identifying which specific components of care most strongly influence maternal mental health outcomes.

## Materials and methods

### Study Setting

We conducted this study in the Upper East Region (UER) of Ghana, which comprises 15 administrative municipalities/districts. Healthcare in the region is provided through 11 district hospitals, 67 health centers, and 419 community-based health planning and services (CHPS) zones, with a regional hospital serving as the primary referral facility [45, 46]. The region faces significant healthcare workforce challenges, with approximately 4 doctors and 200 nurses per 100,000 population, translating to doctor-patient and nurse-patient ratios of 1:25,000 and 1:500, respectively [47].

### Study design and participants

This study was part of a larger study in Ghana that aimed to develop and validate a PCANC scale. This included a cross-sectional survey of 600 women: 300 currently pregnant and 300 within six months postpartum who had received antenatal care during their pregnancy. We used only data from the post-partum sample (*n* = 300) for this analysis, since the postnatal depression questions were only asked of this sample.Data collection occurred during August and September 2023. Since this was part of a validation study, the sample size was estimated based on scale development guidelines recommending 5–10 subjects per scale item [48, 49].

### Data collection

Trained research assistants identified eligible women who had just delivered through facility delivery registers and women who engaged in postnatal care visits. Using convenience sampling, participants were consecutively recruited until reaching the target sample size. One-on-one interviews were conducted in preferred languages of participants’ at preferred times and locations, with responses captured electronically using the REDCap software [50].

### Ethical considerations

The study protocol received approval from the University of California, San Francisco (UCSF) and the Navrongo Health Research Center (NHRC) Institutional Review Boards. All participants provided written informed consent prior to participation. Participants received modest compensation (two cakes of soap) in recognition of their time and contribution.

### Dependent variable: perinatal depression (PND)

The Edinburgh Postnatal Depression Scale (EPDS) was used to assess postpartum depressive symptoms among participants. The EPDS is a widely validated 10-item self-report screening tool that measures emotional and cognitive symptoms of postpartum depression experienced in the past seven days. Each item is scored on a 4-point Likert scale ranging from 0 to 3, yielding a total possible score of 0–30, with higher scores indicating more severe depressive symptomatology. In line with established literature and recommendations from the Postpartum Depression: Action Towards Causes and Treatment (PACT) Consortium (2015), a threshold score of ≥ 10 was used to identify women with significant depressive symptoms, which has also been used in Ghana previously [23]. This cut-off point acknowledges that the scale measures symptom severity rather than providing a clinical diagnosis.

### Independent variables

*The Person-Centered Antenatal Care (PCANC) scale* was used to assess women’s experiences of care during pregnancy. This 36-item validated instrument, which was developed as part of this study, comprises three subscales measuring: dignity and respect (8 items, including privacy and confidentiality of health information), communication and autonomy (15 items, including information provision and consenting), and responsive and supportive care (13 items, addressing aspects like optimal care provision for mother and baby including emotional and mental support). Participants evaluated their antenatal care experiences with healthcare providers during their most recent pregnancy across all dimensions. Each item was scored on a four-point scale ranging from “No, never (or none of them)” [1] to “Yes, all the time (or all of them)” [4], and items were summed to create a score, which was standardized to range from 0 to 100, with higher scores indicating more person-centered care experiences during antenatal period.

*The Person-centered maternity care (PCMC) scale* is a 30-item validated scale administered to postpartum women. The scale evaluates the same three domains of care: dignity and respect (6 items), communication and autonomy (9 items), and supportive care (15 items), with related sets of questions and response options as the PCANC scale. Items for each scale are summed to create a score, which is standardized to range from 0 to 100, where higher scores indicate more person-centered care during childbirth.

### Analysis

All data were retrieved from RedCAP and imported into Stata 18 for cleaning and analysis. Analysis involved descriptive statistics and bivariate analyses to examine associations between the independent variables and PND. We selected potential confounders based on prior research, clinical relevance, and their relationship with both exposure (person-centered care) and outcome (postpartum depression).

We first examined differences in proportions across categories of potential predictors using unadjusted logistic regression clustering standard errors at the facility level. We constructed two separate multivariate logistic regression models: one examining the association between PCANC and postpartum depression, and another examining PCMC and PND. We accounted for potential intragroup correlation at the facility level using appropriate robust standard error procedures. Variables were selected for multivariable models based on statistical significance (*p* < 0.2) in bivariate analyses [51] and/or theoretical relevance [25, 28]. For some conceptually related variables (e.g., income and wealth), we included only one to avoid multicollinearity. We employed stepwise selection (*p* < 0.05) to develop a parsimonious final model. To validate our final model, we compared a full model containing all confounders with a reduced model containing only selected confounders using likelihood ratio testing. Likelihood ratio testing (χ² [12] = 12.15, *p* = 0.4341) confirmed our reduced model was adequate, with good model fit demonstrated by Hosmer-Lemeshow test (χ² [4] = 1.73, *p* = 0.7845) and linktest assessment.

Following the analysis of total scores, we applied the same modeling approach to examine the associations between each of the three subscales for PCANC and PCMC (dignity and respect, communication and autonomy, and responsive and supportive care) and PND. Adjusted odds ratios with 95% confidence intervals were estimated, and a p-value of less than 0.05 was considered significant. We conducted sensitivity analysis using the continuous EPDS score as the outcome variable in linear regression models while maintaining the same covariates and robust standard error procedures to account for facilitylevel clustering. We conducted sensitivity analyses excluding mental health-specific items from the Responsive and Supportive Care subscale of PCANC scale to address potential construct overlap between care measures and depression outcomes.

## Results

Table 1 describes the demographics of the study population (*n* = 293). Participants were predominantly young, with vast majority (97.3%) under the age of 40 years of age. Majority of the participants were married (92.5%) and had varying levels of education with 66.9% having completed at least secondary education. Approximately 40% reported high literacy levels, while 24.9% could not read or write. Most women (70.9%) did not work for pay, with common occupations including housewife/unemployed (24.2%), hairdressing (22.5%), and trading/selling (19.5%). Nearly all participants (98.3%) had health insurance. The majority (62.8%) had given birth once or twice. In terms of antenatal care utilization, only a small minority (6.1%, *n* = 18) received fewer than 4 ANC visits, which is below the World Health Organization’s previous minimum recommendation of 4 visits for uncomplicated pregnancies. Almost half of all women (49.5%) received 8 or more visits, aligning with WHO’s more recent recommendations suggesting 8 contacts during pregnancy for optimal outcomes. Nearly 82% initiating care within the first four months of pregnancy.

**Table 1:** Sample demographics of postpartum women who participated in the cross-sectional survey in Ghana (*N* = 293)

Participant characteristics	*n*	% or mean (SD)
Age categorical (in years)		
20 or Less than 20	41	13.9
21–39	251	83.4
40 or more	8	2.7
Marital status		
Unmarried/Not married	22	7.5
Married	271	92.5
Highest grade completed		
None	15	5.1
Primary or less	53	18.1
Post-primary/vocational	102	34.8
Secondary	94	32.1
College/University	29	9.9
Literacy		
No, cannot read and write	73	24.9
Yes, but with some difficulty with reading	104	35.5
Yes, can read and write very well	116	39.6
Work for pay		
Not worked for pay	208	70.9
Worked for pay	85	29.1
Occupation		
Farming	49	16.7
Trading/selling	57	19.5
Hairdressing	66	22.5
Housewife/unemployed	71	24.2
Teacher/student	24	8.2
Others	26	8.9
Has health insurance		
No	5	1.7
Yes	288	98.3
Parity		
0–2	184	62.8
3	53	18.1
4	33	11.3
5 or more	23	7.8
Number of times received ANC		
Less than 4	18	6.10
4–7	130	44.4
8 or more	145	49.5
Months when you first received ANC for this pregnancy		
2 months or less	115	39.3
3–4 months	125	42.7
5–6 months	42	14.3
7 or more	11	3.7
Pregnancy complication		
No	203	69.3
Yes	90	30.7
Type of delivery		
Vaginal	244	83.3
C-section	49	16.7
Months postpartum		
0–2 months	156	53.3
3–6 months	137	46.7
Income		
None/Undisclosed	30	10.2
100 or less	98	33.5
101–200	58	19.8
201–300	37	12.6
301–400	15	5.1
401–500	20	6.8
More than 500	35	12.0
Tribe		
Kasem	111	37.9
Nankani/Frafra	137	46.8
Builsa	34	11.6
Others	11	3.8
Wealth		
Poorest	58	19.8
Poor	64	21.8
Middle	73	24.9
Rich	49	16.7
Richest	49	16.7

Table 2 presents the summary scores for person-centered care measures and perinatal depression. Both PCANC And PCMC have similar total mean scores (79.1 and 79.5 respectively) though PCMC shows slightly more variability (SD 12.2) compared to PCANC (SD 11.4). Dignity and respect subscale was the domain with the highest score in both scales while communication and autonomy scored the lowest. Both scales show good scoring range, suggesting they can discriminate between different levels of care quality. Regarding perinatal depression, the mean Edinburgh Postnatal Depression Scale (EPDS) score was 4.5 (SD = 5.7, range: 0–22), with 19.8% (*n* = 58) of participants screening positive for probable depression using the EPDS cutoff score of ≥ 10.

**Table 2:** Summary scores for exposures (person-centered antenatal care and person-centered maternity care) and outcomes (*n* = 293)

	Mean (SD)	Min	Max
Person-centered antenatal care (36 items)^			
Total score	79.1 (11.4)	33.3	96.3
Subscales			
Dignity and respect (8 items)	87.5 (13.8)	41.7	100
Communication and autonomy (15 items)	76.0 (14.2)	15.6	100
Responsive and supportive care (13 items)	77.5 (11.6)	30.8	100
Person-centered maternity care (30 items)^^^			
Total score	79.5 (12.2)	31.1	98.9
Subscales			
Dignity and respect (6 items)	90.3 (14.4)	16.7	100
Communication and autonomy (9 items)	75.9 (16.2)	18.5	100
Responsive and supportive care (15 items)	77.3 (13.0)	17.8	97.8
Postpartum depression (10 items)^$^			
Mean and SD of postpartum depression scores	4.5 (5.7)	0	22
Number and prevalence of those who screened positive for depression (EPDS > = 10)	58 (19.8%)		

^^^Higher scores indicate greater person-centered care both in antenatal and maternity scales that are standardized to range from 0 to 100

^$^Higher scores indicate poor mental health or depression

Table 3 shows results from bivariate and multivariable logistic regression analyses between person-centered care scores and PND. In bivariate analyses, each one-point increase in the PCANC scores was associated with 3% lower odds of PND (OR = 0.97, 95% CI: 0.95, 0.99). PCANC’s dignity/respect (OR = 0.97, 95% CI: 0.95, 0.98) and responsive and supportive care (OR = 0.97, 95% CI: 0.95, 0.99) were both significantly associated with lower odds of PND, while the communication and autonomy subscale showed a trend toward protection but was not statistically significant (OR = 0.98, 95% CI: 0.96, 1.00). After adjusting for confounders, the relationship became slightly stronger–– each 1-point increase in PCANC score was associated with 4% lower odds of postpartum depression (aOR: 0.96, 95% CI: 0.93, 0.99). In the adjusted subscale models, both dignity and respect (aOR: 0.96, 95% CI: 0.94, 0.98) and responsive and supportive care (aOR: 0.96, 95% CI: 0.93, 0.99) maintained significant protective associations, while communication and autonomy showed no significant association (aOR: 0.98, 95% CI: 0.95, 1.01).

Higher PCMC scale and sub-scale scores also showed similar protective trends against PND, though associations were not statistically significant at *p* < 0.05 except the dignity and respect subscales, which was significantly associated with lower odds of PND (OR = 0.97, 95% CI: 0.96, 0.99).Communication and autonomy (OR = 0.99, 95% CI: 0.97, 1.00) and responsive and supportive care (OR = 0.98, 95% CI: 0.96, 1.00) showed trends toward protection but were not statistically significant. After adjustment, the total PCMC score (aOR: 0.97, 95% CI: 0.95, 1.00) was not statistically significant with PND. In the adjusted subscale models, only dignity and respect maintained a significant protective association (aOR: 0.97, 95% CI: 0.95, 0.99), while communication and autonomy (aOR: 0.99, 95% CI: 0.96, 1.01) and responsive and supportive care (aOR: 0.98, 95% CI: 0.95, 1.01) showed no significant associations.

**Table 3:** Multivariable logistic regression examining the relationship between person-centered antenatal care, person-centered maternity care and postpartum depression (*n* = 293)

	Unadjusted OR	Adjusted OR
	OR (95%CI)	aOR (95% CI)
Person-centered antenatal care (PCANC)		
Total score	0.97 (0.95, 0.99)*	0.96 (0.93, 0.99)*
PCANC subscales		
Dignity and respect	0.97 (0.95, 0.98)***	0.96 (0.94, 0.98)***
Communication and autonomy	0.98 (0.96, 1.00)	0.98 (0.95, 1.01)
Responsive and supportive care	0.97 (0.95, 0.99)*	0.96 (0.93, 0.99)*
Person-centered maternity care (PCMC)		
Total score	0.98 (0.95, 1.00)	0.97 (0.95, 1.00)
PCMC subscales		
Dignity and respect	0.97 (0.96, 0.99)*	0.97 (0.95, 0.99)*
Communication and autonomy	0.99 (0.97, 1.00)	0.99 (0.96, 1.01)
Responsive and supportive care	0.98 (0.96, 1.00)	0.98 (0.95, 1.01)

Models were adjusted for age, education, wealth index, tribe, month of initiation of ANC, parity, pregnancy complications, had a c-section delivery, and months postpartum

Each adjusted model accounts for potential intragroup correlation at the facility level by using appropriate robust standard error procedures.Data are presented as adjusted odds ratio (aAOR; 95% confidence interval)

*p<0.05, **p<0.01, ***p<0.001

**Fig. 1:**
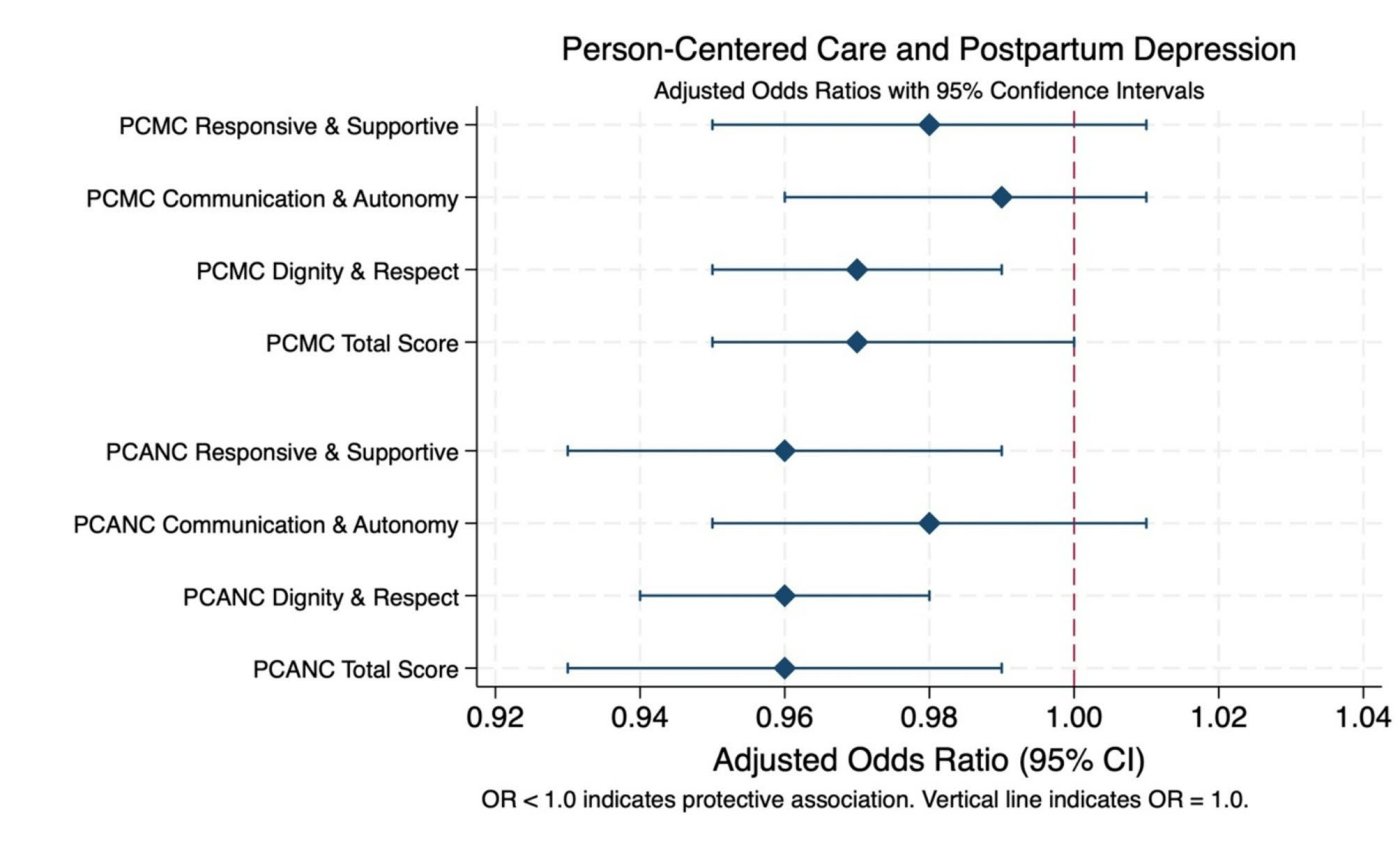
Coefficient plot showcasing the association between person-centered care and perinatal depression

To facilitate interpretation of our main findings, Fig. 1 illustrates the adjusted associations between person-centered antenatal care (PCANC) and person-centered maternity care (PCMC) components with postpartum depression, presented as odds ratios with 95% confidence intervals:

Sensitivity analyses using continuous EPDS scores as the outcome variable produced consistent results with our primary analyses using the dichotomized EPDS score, confirming the robustness of the observed associations between person-centered care measures and postpartum depressive symptoms (results not shown). To address concerns about potential overlap between care measures and depression outcomes, we conducted a sensitivity analysis excluding the two items related to emotional and mental health support from the responsive and supportive care subscale of PCANC scale. The association between this modified subscale and postpartum depression remained significant (aOR: 0.96; 95% CI: 0.93, 0.99; *p* = 0.01), suggesting that the relationship between responsive care and depression is not driven solely by the mental health-specific aspects of care.

## Discussion

### Principal findings

Our study found significant protective associations between PCANC and PND among postpartum women in Ghana. Specifically, higher scores in dignity and respect and responsive and supportive care during antenatal care were associated with lower odds of PND per point increase. For PCMC, only the dignity and respect dimension showed a significant protective association (3% lower odds per point increase), while overall PCMC showed protective trends that failed to reach statistical significance. Surprisingly, communication and autonomy during both antenatal and maternity care showed no significant associations with postpartum depression.

### Results in context

Our study is the first to examine associations between PCANC and PND, providing novel evidence for the potential protective role of respectful, responsive care during pregnancy. While our findings between PCMC and PND were not statistically significant (except for the dignity and respect subscale), they directionally align with previous research examining links between care quality during childbirth and maternal mental health outcomes. In Nepal, higher PCMC was associated with lower depressive symptom scores and improved mental well-being during the postpartum period, though the study only examined PCMC scores rather than individual subscales [52]. Similarly, a longitudinal study of over 1,000 women in Kenya found that PCMC was protective against postpartum depression [28]. Conversely, evidence from a large hospital-based survey of over 23,000 women in Brazil demonstrated that disrespectful and abusive care during childbirth was associated with an increased risk of postpartum depression [53]. Our study extends this literature by examining both antenatal and maternity care, suggesting that the timing and continuity of person-centered care during the perinatal period may be important considerations for PND.

Surprisingly, communication and autonomy during both antenatal and maternity care showed no significant associations with postpartum depression in our study. This finding warrants careful consideration as it contrasts with the theoretical framework underlying person-centered care, which emphasizes the importance of information sharing, shared decision-making, and respecting women’s autonomy in healthcare encounters. However, our null findings may be understood in the context of consistently low communication and autonomy scores across studies using the PCMC scale. A recent systematic review of PCMC studies globally identified communication and autonomy as having ‘the most significant gaps’ among all PCMC domains, highlighting the need to improve PCMC ‘particularly in improving communication and autonomy’ and noting that implementation of effective interventions to improve interpersonal communication and respect for women’s autonomy is especially needed [54]. This shows that if the communication and autonomy domain is universally poor, there may have been insufficient variation in our study as well to detect mental health associations. Our null findings for communication and autonomy align with evidence from a Kenyan study suggesting these associations may be outcome-specific. A study examining PCMC and maternal-newborn health outcomes found that while all three PCMC sub-domains, including communication and autonomy, were associated with women’s intentions for future care-seeking, there was no significant association with newborn immunizations. The authors noted that “other factors beyond women’s experiences, such as access to care or geographical proximity to the facility, may be more important” for certain health outcomes, suggesting that communication and autonomy may influence some outcomes but not others, depending on the underlying mechanisms involved [55]. Further supporting the outcome-specific nature of communication and autonomy associations, research examining PCMC and maternal and newborn health outcomes found that communication and autonomy were significantly associated with behavioral health outcomes such as postpartum family planning uptake and newborn immunizations, but not with maternal complications. The authors suggested that ‘providers engaging women in their healthcare decisions and providing health information are likely to influence their autonomy to obtain health services,’ while maternal complications were more influenced by supportive care and health infrastructure [28].This suggests that communication and autonomy may be particularly important for future health-seeking behaviors that require active decision-making and follow-through, while mental health outcomes such as postpartum depression may be more influenced by the emotional and psychological support provided through dignity, respect, and responsive care during the immediate perinatal period.

Our findings, combined with the global evidence of consistently poor communication and autonomy scores, suggest that this domain requires targeted interventions before its mental health benefits can be realized, particularly in settings like Ghana where these interventions have not yet been systematically implemented. The systematic review noted that low levels of communication and autonomy could affect rapport between healthcare providers and women regarding decision-making, potentially decreasing quality of care. Also, in healthcare systems where hierarchical provider-patient relationships are normative, communication and autonomy may be less salient for mental health outcomes.

### Clinical implications

These findings suggest that person-centered care approaches, particularly focusing on dignity, respect, and responsive care during antenatal visits, could be an important strategy not only for a positive birth experience [56] but also for preventing PND. Person-centered care may work through multiple pathways to impact maternal mental health: building favorable relationships between women and healthcare providers [57], improving women’s self-efficacy [58] and trust in the health system, and encouraging women to seek care when they encounter health problems after delivery. Providers and nurses should be trained not only in clinical skills but also in providing respectful, dignified care during the complete perinatal period, with particular attention to the antenatal period where our findings showed the strongest protective associations.

### Research implications

Longitudinal and mediation studies are needed to establish causality and to explore the pathways through which person-centered care during the antenatal and maternity period is associated with lower PND [59]. Future research should examine whether targeted interventions to improve communication and autonomy can demonstrate mental health benefits and investigate cultural factors influencing how these domains are experienced in different contexts. Additionally, research should explore the specific mechanisms through which dignity, respect, and supportive care protect against postpartum depression to inform intervention development. However, given the broader importance of PCMHC, these findings support the need for intervention studies to determine the most effective approaches for improving person-centered care. Further, there is a need for integrated approaches to improve person-centered care that address the mental wellbeing of mothers as well as providers [60]. Ongoing studies such as the ‘Caring for Providers to Improve Patient Experience (CPIPE)’ trial in Kenya and Ghana [61] seek to extend this evidence.

### Strengths and limitations

Our study has some limitations. The design was cross-sectional, which precludes any causal inference. The reliance on self-reported data introduces potential recall and social desirability bias, particularly for healthcare experiences and depression screening responses. An important limitation of our study is that we focused exclusively on postpartum women rather than including the full sample of 600 women (300 pregnant and 300 postpartum) from our larger validation study. The EPDS was not administered to women in the antenatal phase of our data collection, which prevented us from examining prenatal depression and its relationship with person-centered care. This represents a significant missed opportunity, as women with depressive symptoms during pregnancy often have higher rates of PND, and assessing both antenatal and postnatal mental health would have provided a more comprehensive understanding of the perinatal mental health continuum. Our study’s geographic scope was restricted to a single-region in Ghana, with convenience sampling from specific facilities, limiting generalizability to other contexts. Additionally, the timing of depression screening varied across participants (ranging from immediate postpartum to six months after delivery) could have influenced our results, as women’s mental health experiences may differ across the postpartum period. Nonetheless, a significant strength of our study includes using the validated measures of person-centered care during antenatal and childbirth and their subscales to reveal insights into potential mechanisms between person-centered care and PND. Our study’s focus on both antenatal and maternity care provides unique insights into the relative importance of care quality at different time points.

## Conclusion

This study provides novel evidence that PCANC is significantly associated with lower odds of PND, with dignity and respect and responsive and supportive care being important components of care. While we did not find a significant association between PCMC and PND except for dignity and respect subscale, person-centered approaches integrated into healthcare provision can prevent PND and improve women’s mental health and well-being. These findings suggest that strengthening person-centered care during pregnancy could be a promising strategy for addressing maternal mental health in low- and middle-income settings, especially where routine screening of depression is not available.

## Supplementary Information

Supplementary Material 1.

## Data Availability

The dataset for this study can be made available upon reasonable request to the corresponding author.
