# Supplementary material for: Association between person-centered care during pregnancy and perinatal depression in Ghana
Source: BMC Pregnancy Childbirth. 2025 Oct 6;25:1021. doi: 10.1186/s12884-025-07966-6 (PMC12502305; doi:10.1186/s12884-025-07966-6)
Supplement: Supplementary file 1 — Supplementary Material 1. [file 12884_2025_7966_MOESM1_ESM.docx]

**Supplementary Table 1**. **Bivariate regression analyses to examine factors related to perinatal depression among 293 postpartum women in Ghana.**

| **Participant characteristics** | **n** | **% or mean (SD)** | **Unadjusted odds ratio (95% CI)** | **p-value** |
| --- | --- | --- | --- | --- |
| **Age (years), mean/SD** | 293 | 26.8 (5.8) | 1.05 (0.98, 1.13) | 0.065^#^ |
| **Marital status** |  |  |  |  |
| Unmarried/Not married | 22 | 7.5 |  |  |
| Married | 271 | 92.5 | 0.82 (0.29, 2.33) | 0.720 |
| **Highest grade completed** |  |  |  |  |
| None | 15 | 5.1 |  |  |
| Primary or less | 53 | 18.1 | 0.44 (0.14, 1.30) | 0.138^#^ |
| Post-primary/vocational | 102 | 34.8 | 0.41 (0.13, 1.30) | 0.131^#^ |
| Secondary | 94 | 32.1 | 0.24 (0.06, 0.83) | 0.024^#^ |
| College/University | 29 | 9.9 | 0.31 (0.06, 1.57) | 0.159^#^ |
| **Literacy** |  |  |  |  |
| No, cannot read and write | 73 | 24.9 |  |  |
| Yes, but with some difficulty with read | 104 | 35.5 | 0.63 (0.32, 1.20) | 0.164^#^ |
| Yes, can read and write very well | 116 | 39.6 | 0.48 (0.25, 0.96) | 0.039^#^ |
| **Work for pay** |  |  |  |  |
| Not worked for pay | 208 | 70.9 |  |  |
| Worked for pay | 85 | 29.1 | 0.98 (0.35, 2.74) | 0.977 |
|  |  |  |  |  |
| **Occupation** |  |  |  |  |
| Farming | 49 | 16.7 |  |  |
| Trading/selling | 57 | 19.5 | 0.73 (0.21, 2.46) | 0.613 |
| Hairdressing | 66 | 22.5 | 0.96 (0.38, 2.37) | 0.924 |
| Housewife/unemployed | 71 | 24.2 | 1.42 (0.57, 3.55) | 0.447 |
| Teacher/student | 24 | 8.2 | 1.03 (0.32, 3.21) | 0.964 |
| Others | 26 | 8.9 | 0.32 (0.07, 1.54) | 0.158^#^ |
| **Has health insurance** |  |  |  |  |
| No | 5 | 1.7 |  |  |
| Yes | 288 | 98.3 | 0.98 (0.11, 8.76) | 0.991 |
| **Parity** |  |  |  |  |
| 0-2 | 184 | 62.8 |  |  |
| 3 | 53 | 18.1 | 1.39 (0.67, 2.85) | 0.371 |
| 4 | 33 | 11.3 | 1.05 (0.42, 2.66) | 0.909 |
| 5 or more | 23 | 7.8 | 2.53 (1.00, 6.37) | 0.048^#^ |
| **Number of times received ANC** |  |  |  |  |
| Less than 4 | 18 | 6.10 |  |  |
| 4-7 | 130 | 44.4 | 0.96 (0.33, 2.77) | 0.941 |
| 8 or more | 145 | 49.5 | 0.76 (0.24, 2.34) | 0.639 |
| **Months when you first received ANC for this pregnancy** |  |  |  |  |
| 2 months or less | 115 | 39.3 |  |  |
| 3-4 months | 125 | 42.7 | 0.99 (0.52, 1.89) | 0.990 |
| 5-6 months | 42 | 14.3 | 0.63 (0.23, 1.69) | 0.361 |
| 7 or more | 11 | 3.7 | 0.84 (0.19, 3.68) | 0.820 |
| **Pregnancy complication** |  |  |  |  |
| No | 203 | 69.3 |  |  |
| Yes | 90 | 30.7 | 1.50 (0.81, 2.75) | 0.191^#^ |
| **Type of delivery** |  |  |  |  |
| Vaginal | 244 | 83.3 |  |  |
| C-section | 49 | 16.7 | 2.33 (1.18, 4.60) | 0.015^#^ |
| **Months postpartum** |  |  |  |  |
| 0-2 months | 156 | 53.3 |  |  |
| 3-6 months | 137 | 46.7 | 1.81 (1.09, 3.02) | 0.022^#^ |
| **Household characteristics** | | | | |
| **Income** |  |  |  |  |
| None/Undisclosed | 30 | 10.2 |  |  |
| 100 or less | 98 | 33.5 | 0.59 (0.26, 1.39) | 0.232 |
| 101- 200 | 58 | 19.8 | 0.42 (0.16, 1.15) | 0.094^#^ |
| 201- 300 | 37 | 12.6 | 0.28 (0.07, 1.03) | 0.057^#^ |
| 301- 400 | 15 | 5.1 | 0.35 (0.07, 1.82) | 0.217 |
| 401- 500 | 20 | 6.8 | 1.55 (0.42, 5.74) | 0.508 |
| More than 500 | 35 | 12.0 | 0.48 (0.14, 1.65) | 0.248 |
| **Tribe** |  |  |  |  |
| Kasem | 111 | 37.9 |  |  |
| Nankani/Frafra | 137 | 46.8 | 1.28 (0.64, 2.56) | 0.480 |
| Builsa | 34 | 11.6 | 0.25 (0.05, 1.24) | 0.091^#^ |
| Others | 11 | 3.8 | 0.40 (0.05, 3.51) | 0.388 |
| **Wealth** |  |  |  |  |
| Poorest | 58 | 19.8 |  |  |
| Poor | 64 | 21.8 | 0.67 (0.28, 1.61) | 0.377 |
| Middle | 73 | 24.9 | 0.47 (0.21, 0.07) | 0.074^#^ |
| Rich | 49 | 16.7 | 0.37 (0.11, 1.03) | 0.059^#^ |
| Richest | 49 | 16.7 | 0.54 (0.19, 1.47) | 0.232 |

Note: Only coefficients significant at p<0.2 level are highlighted with ^#^
